# Circular statistics meets practical limitations: a simulation-based Rao’s spacing test for non-continuous data

**DOI:** 10.1186/s40462-019-0160-x

**Published:** 2019-05-10

**Authors:** Lukas Landler, Graeme D. Ruxton, E. Pascal Malkemper

**Affiliations:** 1 Research Institute of Molecular Pathology (IMP), Vienna Biocenter (VBC), Campus-Vienna-Biocenter 1, 1030 Vienna, Austria; 2 School of Biology, University of St Andrews, St Andrews, KY16 9TH UK

**Keywords:** Circular statistics, Limited precision, Rayleigh test, Testing for circular uniformity, Randomisation testing, Statistical power, Oriana

## Abstract

**Background:**

For data collected on a circular rather than linear scale, a very common procedure is to test whether the underlying distribution appears to deviate from circular uniformity. Rao’s spacing test is often used to evaluate the support the data offers for the null hypothesis of uniformity. Here we demonstrate that the traditional version of this test fails to adequately control type I error rate when the data is non-continuous (i.e. is rounded/grouped to a finite number of discrete values, e.g. to the nearest degree, a common situation). To overcome this issue, we provide a numerically-intensive simulation version of the test.

**Methods:**

We use a simulation study to explore the performance of the traditional and our novel variant on Rao’s spacing test, both in terms of control of type I error rate and statistical power.

**Results:**

When data is measured on a continuous circular scale then both methods offer good control of type I error and similar statistical power. If the data is rounded (even to a relatively fine scale such as to the nearest degree – giving 360 possible values), however, the traditional method produces highly inflated type I error rates, particularly with high sample sizes, that make it inappropriate for application to such data. In contrast, our simulation method retains good control of type I error while offering levels of statistical power similar to the traditional Rao test.

**Conclusions:**

The traditional method of applying Rao’s spacing test should be replaced by the simulation-based variant introduced here. The two methods offer similar performance but only the simulation method retains good control of the type I error rate when circular data is rounded to a finite set of values (likely due to limited precision of measuring equipment). Adoption of the simulation variant will substantially improve the reliability of this regularly-used test in the commonplace situation where data values are rounded.

**Electronic supplementary material:**

The online version of this article (10.1186/s40462-019-0160-x) contains supplementary material, which is available to authorized users.

## Background

Circular data is commonly generated whenever researchers measure angles (such as the distribution of compass directions that seeds disperse from a parent plant) or have hypotheses related to cyclic behaviour (e.g. looking for variation in rate of animal movement over the daily cycle). Such data needs different treatment to data measured on linear scales, and a number of monographs are available on the statistical treatment of circular data (e.g. Batschelet [1]; Fisher [2]; Jammalamadaka and SenGupta [4]; Mardia and Jupp [7]; Pewsey, Neuhäuser, and Ruxton [8]; Ley and Verdebout [6]). The most common procedure in circular statistics is testing to see if a sample of values suggests deviation from being uniformly distributed around the circle. If any deviation is expected to be unimodal then the most commonly-applied test for this is the Rayleigh test (introduced by Lord Rayleigh in 1880 [9], but also described in the monographs listed previously). If, however, the deviation might have more than one mode (e.g. when several dispersal directions or activity periods are expected) the power of the Rayleigh test to detect these deviations is low. A number of alternatives are available – and the Rao spacing test [11–13] is one commonly recommended (e.g. Batschelet [1]; Fisher [2]; Jammalamadaka and SenGupta [4]). In a recent comparison of the power of different non-Rayleigh alternative tests in circular statistics, Rao’s test performed well [5], specifically in multimodal cases when the modes are distributed symmetrically around the circle. The Rao spacing test may also be preferred when the shape of the potential deviation might be expected to deviate from the symmetric bell-shaped von Mises distribution on which the Rayleigh test is based, because the theoretical underpinning of the Rao spacing test makes no assumption about the potential shape of any deviation from uniformity. For these reasons Rao’s spacing test remains in common use. The test is based on the simple idea that if *n* individual data points are distributed perfectly uniformly around a circle then the length of arc between neighbouring points is simply 2π/*n* in radian measure or 360/*n* in degrees. The test statistic is based on the sum of the deviations of the actual arc lengths from this expectation, and a sufficiently high test statistic suggests departure from uniformity.

One attraction of this test is its universal applicability, since it rests on no particular assumption about the nature of any departure from normality, unlike many alternative tests [4] and it has been shown to have good power compared to alternative tests in some circumstances (e.g. when sample sizes are low: [11]). However, here we highlight a rarely-discussed but significant limitation to the test in its traditional form, and simple procedures that we demonstrate overcome this limitation.

## Methods

### Defining the test

We define the test for *n* observations *ϕ*_*1*_, …,*ϕ*_n_ taken in radian measure so as to lie within [0,2*π*). We further assume that the observations are ordered from smallest to largest: *ϕ*_*1*_ < *ϕ*_*2*_ < …. < *ϕ*_*n-1*_ < *ϕ*_*n*_. We can then define the set of *n* arc lengths between neighbouring points as

$$ {T}_i=\left\{\begin{array}{c}{\phi}_{i+1}-{\phi}_i, if\ i<n\\ {}2\pi -{\phi}_n+{\phi}_1\  if\ i=n\end{array}\right. $$

The test statistic *U* is then the sum of the differences between the actual arc lengths and their expected values under uniformity (2*π*/*n*).

$$ U=0.5\sum \limits_{i=1}^n\left|{T}_i-\frac{2\pi }{n}\right| $$

If *U* exceeds a critical value then the null hypothesis of uniformity is rejected. Based on the moments of the underlying distribution, Russell & Levitin [10] provide critical values for alpha = 0.001, 0.05, 0.01, 0.5, 0.1, 0.5 and 0.9 for every sample size from 4 to 30, and for a selection of higher values: 35, 40, 45, 50, 75, 100, 150, 200, 300, 400, 500, 600, 700, 800, 900, 1000. These values are used by the R function *rao.spacing.test* in the package *circular*, and all implementations of the test we have seen in the literature (see [1] for some examples) use the traditional approach of comparing calculated values against these critical values. Alternatively, the *p*-value can be estimated by simulation, where the p-value is the fraction of samples drawn from a uniform distribution that have a *U* value higher than the observed one. Here we argue for greater use of this second method.

To obtain a p-value for this test by simulation the following approach was taken for continuously-distributed data. First, the value of the test statistic is obtained for the observed sample (call this value *U*_*o*_). We then generate *N*_*R*_ samples from a continuous uniform distribution, each with the same sample size as the original sample. For each of these simulated samples we calculate the value of the test statistic, and calculate the number *N*_*e*_ of these simulated samples that produce a test statistic value equal to or greater than *U*_*o*_. The estimated *p* value is then (*N*_*e*_+ 1)/(*N*_*R*_ + 1). In our simulations we used *N*_*R*_ = 10,000. In the case of discrete (rounded) data, this approach needs to be modified because the restrained number of options the random distribution can be drawn from (e.g. 360 when rounded to one degree) would otherwise create many identical distributions. To obtain a *p*-value for discrete (rounded) data by simulation the following approach was taken. First, we added very small random perturbations selected independently from a von Mises distribution with mean zero to each data-point in our sample. That is for data point *ϕ*_*i*_ we obtain a perturbation *ε*_*i*_ drawn from a von Mises with mean zero and with concentration parameter *κ*. We then calculate the value of the test statistics for the observed sample with added perturbations (call this value *U*_*o*_). Notice that if the sum of the original value and perturbation is outside of [0,2π) then we add or subtract 2π (modulo 360) as required to correct this prior to calculating the test statistic. We then generate *N*_*R*_ samples from a continuous uniform distribution, each with the same sample size as the original sample. For each of these simulated samples we (i) round values to the same precision as the original sample, then (ii) add perturbations in the same way as we did for the original sample (again correcting to make sure that the perturbed values still lie in [0,2π)), and then finally (iii) calculate the value of the test statistic for each perturbed simulated sample. We then calculate the number *N*_*e*_ of these perturbed simulated samples that produce a test statistic value equal to or greater than *U*_*o*_. The estimated *p* value is then (*N*_*e*_+ 1)/(*N*_*R*_ + 1). In our simulations we used *N*_*R*_ = 10,000, and *κ* = 1000. The higher the value of *κ* the more concentrated the distribution. A value should be chosen that is high enough that the perturbations are much smaller than the granularity of the imprecision. That is, if (for example) original values were obtained to the nearest 10 degrees, then a value of *κ* should be selected to ensure that almost all perturbations are less than 1 degree. See supplementary files for R codes for both simulation-based approaches (Additional file 2; the input sample can be in in radians or degrees and between − 4 and 6*pi or − 720 to + 1080 degrees for the function to work).

### Simulations for type I error

We estimate the type I error rate as the fraction of 10,000 samples each drawn from a uniform distribution that cause the test to generate a *p*-value of less than 0.05. We expect the type I error rate of an effective test to remain close to the nominal value (0.05 in this case). We evaluate the p-value both in the traditional way using the R function *rao.spacing.test* in the package *circular* and using our simulation approach. Because Rao’s spacing test considers the distribution of arcs between data points we might expect it to be sensitive to any rounding of the values due (for example) to finite precision of equipment used to record values. Therefore, we repeated this analysis using circular uniform distributions rounded to the next degree (360 bins) and to the next 10 degrees (36 bins), two situations, due to practical reasons, commonly encountered in biological studies.

### Power comparisons for von Mises and skew normal distributions

We estimated the power of the different approaches to correctly detect deviation from uniform distribution. For this we first generated von Mises (*κ* = 2, μ = pi) and skew normal distributions (ε = pi, α = 30, ω = 2) with continuous data of different sample sizes and calculated the power as the fraction of 10,000 samples that generate a *p*-value equal to or less than 0.05, using both approaches. In a next step we rounded the von Mises distribution to the next degree and also the next 10 degrees and repeated the analysis using the simulation approach.

## Results

**Fig. 1:**
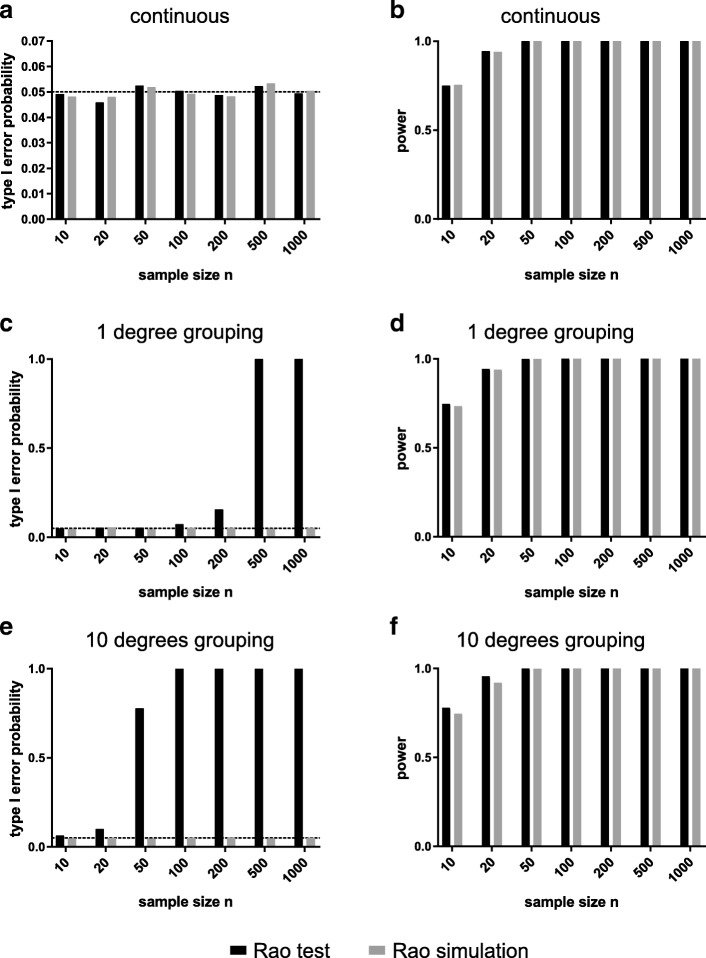
Type I error rates (**a**, **c**, **e**) and power for a von Mises distribution (**b**, **d**, **f**) of the traditional Rao and the simulation-based Rao test with different sample sizes. (**a**, **b**) First, we tested a continuous uniform distribution (type I error) and a von Mises distribution (power), *p*-values are evaluated by the R function rao.spacing.test in the package circular and by simulation, showing similar type I error probabilities and power. (**c**, **d**) Second, we tested simulated data rounded to the next degree (360 bins) and applied either the traditional or the simulation-based test. (**e**, **f**) Then we repeated the type I error and power estimation for data binned in 36 equal bins (rounded to the next 10°). The dashed line indicates the nominal 5% level for type I errors. The traditional Rao test shows inflated type I error rates when used on rounded data (**c**, **e**). The simulation-based test has low type I error rates and offers power similar to the traditional Rao test (**d**, **f**)

We found that control of type I error is generally good for both methods when using samples measured on a continuous circular scale (Fig. 1a). However, when data are rounded to the nearest degree, we can see that type I error rate for the traditional variant remains near the nominal 5% level only for small sample sizes, but for sample sizes greater than 50 begins to climb higher (Fig. 1c). This is an unusual circumstance where the robustness of a test to deviation from its underlying assumptions declines with increasing sample size. This can be seen as being caused by increasing commonness of ties in the data with increasing sample size. In other words the Rao’s spacing test is detecting the ‘clustering’ of 360 modes in the data, essentially showing the power of the test to detect all types of divergence of uniformity. Turning to rounding to the nearest 10 degrees we see the issue of inflation of type I error rate when using the traditional method is a much greater problem than under the previous scenario (Fig. 1e). Here, the type I error rate remains near the nominal 5% level only for the smallest sample size of 10 and drastically increases with larger samples. In contrast, we can see that the simulation methodology offers good control of type I error rate for all sample sizes considered.

Next, we tested the power of the two Rao spacing test variants. The power of the simulation-based approach is almost identical to the traditional method when applying it to continuous data, showing a good performance of our Rao test variation (Fig. 1b for von Mises and Additional file 1: Figure S1 a for skew normal distributions). When data is rounded to the nearest degree or ten degrees prior to evaluation by simulation then power remains largely unchanged (Fig. 1d, f, see Additional file 1: Figure S1 b, c for skew normal distributions). Thus, evaluation of Rao’s spacing test by simulation remains a viable means for testing for departure for uniformity even if the data is grouped, but the traditional method cannot be recommended in this case because of the potential for very serious inflation of type I error rates.

## Discussion

A recent study suggested that rounding to a finite number of values was the norm for recently published papers in the field of behavioural ecology at least [3]. That study found that rounding to 4, 8 and 12 possible values was by no means uncommon, and so the rounding to either 360 or 36 values used here can be considered conservative. The problems with inflation of the type I error rate in the traditional version of the test increase as the number of possible values for data points decreases. Furthermore, the type I error rapidly increases with sample size, leading to situations where users of the traditional test might be very confident about the validity of the test outcome, due to large sample sizes, but they might be deceived by the unusual way the type I error of this test behaves. In many cases, in particular when studying animal behaviour, it is almost impossible to measure angles (with confidence) more precisely than to the next five or ten degrees. Using the traditional Rao test in these cases would mean high risk for false positive findings. Our simulation-based approach corrects for the described issues with the traditional method, while the power remains similar.

However, our simulation results are by no means exhaustive. The robustness of our results could be expanded in several ways: nominal type I error rates other than 5% could be explored, and/or a wider range of sample sizes and levels of rounding for individual data points (e.g. to five degrees). Further, the statistical power of our new method could be explored for a broader range of shapes of deviation from uniformity. Finally, the sensitivity of our method to selection of the size of the perturbations added to data-points could be explored. However, our own preliminary explorations (not shown) give us no reason to expect that the results presented here are unrepresentative. We hope that our simulations provide the basis of further development of circular statistical methods and spark comprehensive evaluation of different methods. False positive results are a large concern in life sciences; our provided functions therefore might help to avoid some unintentional statistical mistakes, when dealing with circular data.

## Conclusion

Here we highlight a critical hitherto overlooked limitation of Rao’s spacing test for departure from circular uniformity. If data is grouped (for example because of finite precision of measurement) then this causes inflation of type I error rates, i.e. the test is detecting the non-uniformity of rounding the data. Such rounding is very common throughout practical science. We show that this inflation of type I error rate can be very substantial even for moderate rounding (to the nearest degree or 10 degrees), and that the problem is exacerbated by large sample sizes. We advocate evaluation by simulation, comparing the sample to a test set of similar samples drawn from a uniform distribution. We demonstrate that this avoids type I error problems and maintains power. We provide *R* functions to perform this test by simulation in the two cases where sample values can be considered continuous or where the data is grouped into a specified number of equal divisions around the circle (Additional file 2). The widespread adoption of these methods would greatly improve the applicability and reliability of Rao’s spacing test to a fundamental hypothesis testing situation for circular data.

## Additional files

Additional file 1: **Figure S1.** Power (a, b, c) of the traditional Rao and the simulation-based Rao test with different sample sizes on a skew normal distribution. (PDF 70 kb)

Additional file 2: R code for the simulation-based Rao test. (R 5 kb)

## References

[1] Batschelet E (1981). Circular statistics in biology.

[2] Fisher NI (1995). Statistical analysis of circular data.

[3] Humphreys RK, Ruxton GD (2017). Consequences of grouped data for testing for departure from circular uniformity. Behav Ecol Sociobiol.

[4] Jammalamadaka SR, SenGupta A (2001). Topics in circular statistics.

[5] Landler L, Ruxton GD, Malkemper EP (2018). Circular data in biology: advice for effectively implementing statistical procedures. Behav Ecol Sociobiol.

[6] Ley C, Verdebout T (2017). Modern directional statistics.

[7] Mardia KV, Jupp PE (2009). Directional statistics.

[8] Pewsey A, Neuhäuser M, Ruxton GD (2013). Circular statistics in R.

[9] Rayleigh L (1880). Xii. On the resultant of a large number of vibrations of the same pitch and of arbitrary phase. Lond.Edinb.Dubl.Phil.Mag..

[10] Russell GS, Levitin DJ (1995). An expanded table of probability values for Rao's Spacing Test. Comm. In Stat.-Simul. & Comput.

[11] Rao JS (1969). Some contributions to the analysis of circular data. Ph.D. thesis.

[12] Rao JS (1972). Some variants of chi-square for testing uniformity on the circle. Z. Wahrscheinlichkeitstheor. verw. Geb.

[13] Rao JS (1976). Some tests based on arc-lengths for the circle. Sankhya: India. J Stat. Ser. B.

