# Supplementary material for: Circular statistics meets practical limitations: a simulation-based Rao’s spacing test for non-continuous data
Source: Mov Ecol. 2019 May 10;7:15. doi: 10.1186/s40462-019-0160-x (PMC6511169; doi:10.1186/s40462-019-0160-x)
Supplement: Supplementary file 1 — Figure S1. Power (a, b, c) of the traditional Rao and the simulation-based Rao test with different sample sizes on a skew normal distribution. (PDF 70 kb) [file 40462_2019_160_MOESM1_ESM.pdf]

Online Resource 1: Supplementary figure

Movement Ecology

Circular statistics meets practical limitations: A simulation-based Rao's spacing test for non-continuous data

Lukas Landler[1], Graeme D. Ruxton[2], E. Pascal Malkemper[1]

Affiliations

1 Research Institute of Molecular Pathology (IMP), Vienna Biocenter (VBC), Austria

2 School of Biology, University of St Andrews, St Andrews KY16 9TH, UK

Corresponding author email address: pascal.malkemper@imp.ac.at

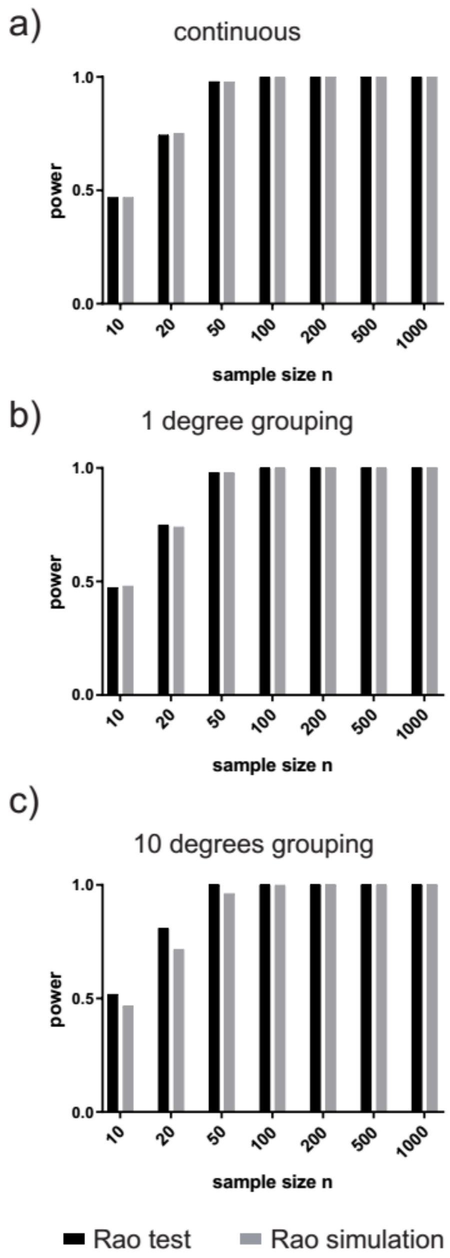

Fig. A1 Power (a, b, c) of the traditional Rao and the simulation-based Rao test with different sample sizes on a skew normal distribution. We tested a continuous uniform distribution; p-values are evaluated by the R function rao.spacing.test in the package circular and by simulation, showing similar power for continuous distributions (a). The simulation approach also retains similar power to the traditional approach using data with 360 bins (b) and 36 bins (c).
